# Diversity of incubation rhythms in a facultatively uniparental shorebird – the Northern Lapwing

**DOI:** 10.1038/s41598-019-41223-z

**Published:** 2019-03-18

**Authors:** Martin Sládeček, Eva Vozabulová, Miroslav E. Šálek, Martin Bulla

**Affiliations:** 1 Faculty of Environmental Sciences, Czech University of Life Sciences Prague, Kamýcká 129, Praha–Suchdol, 165 00 Czech Republic; 2 NIOZ Royal Netherlands Institute for Sea Research, Department of Coastal Systems and Utrecht University, P.O. Box 59, 1790 AB Den Burg, The Netherlands; 3 Department of Behavioural Ecology and Evolutionary Genetics, Max Planck Institute for Ornithology, Eberhard Gwinner Str, 82319 Seewiesen, Germany

## Abstract

In birds, incubation by both parents is a common form of care for eggs. Although the involvement of the two parents may vary dramatically between and within pairs, as well as over the course of the day and breeding season, detailed descriptions of this variation are rare, especially in species with variable male contributions to care. Here, we continuously video-monitored 113 nests of Northern Lapwings *Vanellus vanellus* to reveal the diversity of incubation rhythms and parental involvement, as well as their daily and seasonal variation. We found great between-nest variation in the overall nest attendance (68–94%; median = 87%) and in how much males attended their nests (0–37%; median = 13%). Notably, the less the males attended their nests, the lower was the overall nest attendance, even though females partially compensated for the males’ decrease. Also, despite seasonal environmental trends (e.g. increasing temperature), incubation rhythms changed little over the season and 27-day incubation period. However, as nights shortened with the progressing breeding season, the longest night incubation bout of females shortened too. Importantly, within the 24h-day, nest attendance was highest, incubation bouts longest, exchange gaps shortest and male involvement lowest during the night. Moreover, just after sunrise and before sunset males attended the nest the most. To conclude, we confirm substantial between nest differences in Lapwing male nest attendance, reveal how such differences relates to variation in incubation rhythms, and describe strong circadian incubation rhythms modulated by sunrise and sunset.

## Introduction

A parent incubating eggs is a rare site across the animal kingdom^1,2^, but not so in birds^3^. In the vast majority of avian species, incubating parents actively maintain egg temperatures in a range that is optimal for embryonic development (e.g. by siting, shading or wetting the eggs). Moreover, in almost 50% of bird families incubation by both parents is the most common form of care for eggs^3^. Yet, species vary greatly in how parents divide and time their incubation, that is species vary in their incubation rhythms^4–6^.

In some species, such as seabirds, one parent sits on the nest continuously for several days (e.g. refs^7–9^). In others, one parent sits continuously on the nest for a few hours^10–13^ or even only for few minutes^14^. In some species, both sexes share incubation duties nearly equally^6,13,15^; in others, one sex incubates far more than the other^16–19^, be it in terms of nest attendance^16–19^ and/or incubation efficiency (i.e. incubation temperatures)^16,20,21^. Thus, although the general between-species differences in how parents divide and time their incubation are somewhat known, detailed descriptions over the day and season (i.e. as ambient temperatures and predation pressure change) are uncommon^6,15,22–26^ and often limited to species with incubation bouts lasting several days^7–9^. Moreover, although between- and within-pair differences in incubation rhythms might be considerable^6,13^, and in extreme cases one parent may even desert its incubating partner^24^, detailed analysis of such between- and within-pair differences is often also lacking.

Here, we used continuous video-monitoring of 113 nests to describe the incubation rhythms of the Northern Lapwing *Vanellus vanellus*, a common Palearctic shorebird with variable male contribution to incubation^27–31^. Current knowledge about incubation of Northern Lapwings is mostly based on brief sampling periods of a few hours^27,29–31^(but see ref.^28^). Our continuous data allowed for a detailed description of daily and seasonal variation in how sexes divide their incubation duties between and within pairs. In shorebirds, including our Lapwing population, nest attendance highly correlates with actual incubation (warming or shading of eggs^13,24,32^; Supplementary actograms^33^). Also, females and males seem to heat the cutch to the similar temperatures (^13,24,32,34^; Supplementary actograms^33^).

We specifically investigated (1) between-nest variation in overall nest attendance (proportion of observed time parents were sitting on the nest or shading the eggs), (2) how male nest attendance relates to this between-nest variation in nest attendance, as a single parent cannot incubate with high nest attendance indefinitely, and (3) tested how incubation rhythm (female and male contribution) changed within a day, throughout the incubation period and season as food availability^35,36^, temperature and predation pressure^37,38^, as well as brood value vary over temporal time scales^39–41^.

## Results

### Overall nest attendance

**Figure 1:**
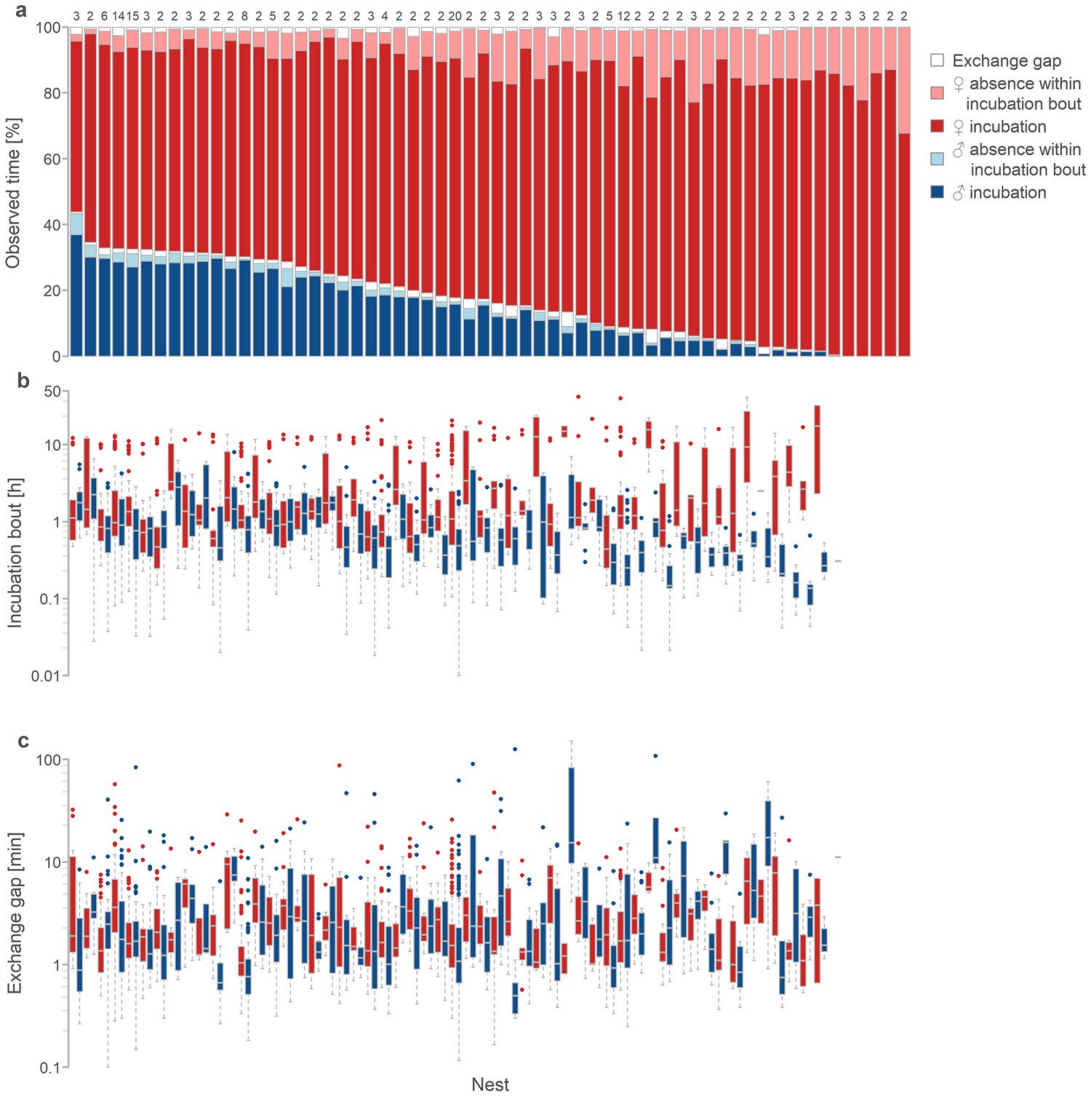
Between and within nest variation in incubation. (**a**) Between- and within-nest variation in nest attendance. Each bar represents one nest and proportion of female incubation bouts (red), male incubation bouts (blue) and exchange gaps (gaps preceding female incubation bouts are above female bars and those preceding male incubation bouts are above male bars). Dark colours indicate actual incubation (individual sitting on the nest) and light colours indicate the absence of a parent (no incubation) within its incubation bouts. Numbers above the bars indicate the number of days with incubation data. Nests (bars) are ordered from the highest to the lowest male nest-attendance (*N* = 60 nests). (**b**,**c**) Between- and within-nest variation in incubation bouts (**b**) and exchange gaps (**c**) according to sex (female in red, male in blue). Each pair of box plots (female and male) corresponds to the nest (bar) in (**a**) (*N* = 2239 bouts and gaps from 55 biparental nests). Box plots depict median (vertical line inside the box), 25–75th percentiles (box), 25th and 75th percentiles minus or plus 1.5× interquartile range, respectively, or the minimum and maximum value, whichever is smaller (whiskers) and outliers (circles). (**a**–**c**) The between nest variation is unlikely driven by when the nest started within the breeding season or within which part of the incubation period it was monitored (Tables S3, S7, S8^33^).

Northern Lapwing parents incubated their eggs 87% of time (median, range: 68–94%, *N* = 60 nests with more than 2 days of recording; Fig. 1a, dark red and blue). Actual incubation bouts, defined as the time between the arrival of a parent at the nest and its departure, followed by the incubation of its partner (i.e. the total time allocated to a single parent including incubation recesses), covered 98% of observed time (median, range: 95–100%, *N* = 55 nests incubated by both parents; Fig. 1a, red and blue). In other words, one parent was nearly always responsible for the nest (sum of incubation bouts). Exchange gaps, defined as the time between the departure of one parent from the nest and the return of its partner, thus accounted for only 2% of observed time (median, range: 0.3–5%, *N* = 55 nests incubated by both parents).

Females incubated more than males because they attended the nest 72% of observed time (median, range: 52–87%, *N* = 60 nests with more than 2 days of recording; Fig. 1a in dark red) and were responsible for the nest, that is their incubation bouts covered, 82% of observed time (median, range: 54–100%; Fig. 1a in dark and light red; note that 100% represents 5 nests incubated solely by females). Females were absent from the nest during their incubation bouts (i.e. incubation recesses covered) 10% of the time (median, range: 2–32%; Fig. 1a in light red). In contrast, males attended the nests 13% of the time (median, range: 0–37%; Fig. 1a in dark blue) and were responsible for the nest 15% of the time (median, range: 0–43%; Fig. 1a in dark and light blue). Recesses during male incubation bouts covered 7% of the time (median, range: 0–22%; *N* = 55 nests with male incubation; Fig. 1a in light blue). Note that overall female nest attendance was always higher than that of males and the two were strongly negatively correlated (*r* = −0.87; Fig. S3a).

**Figure 2:**
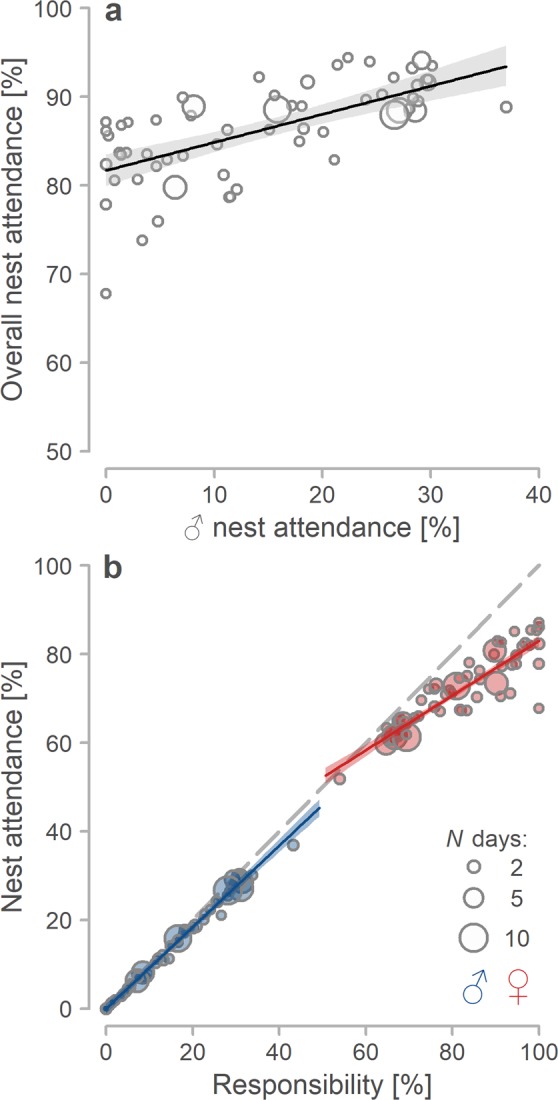
Contribution of females and males to overall nest attendance. (**a**) Relationship between male nest attendance and overall nest attendance (*N* = 60 nests). (**b**) Relationship between responsibility (proportion of all parent’s incubation bouts within observed time) and nest attendance for females (red) and males (blue; *N* = 120 parents from 55 biparental nests). (**a**,**b**) Circles represent individual nests (**a**) or parents (**b**) and their size, the number of days with incubation data. Lines with shaded areas indicate model prediction with 95% credible intervals based on the joint posterior distribution of 5,000 simulated values based on model outputs (Tables S1 and S2^33^) and generated by the ‘sim’ function in R^90^. Included are only nests with at least two days of incubation data and days with at least 90% of recording. The dashed line in **b** indicates full compensation for the reduced care of a partner.

Overall nest attendance decreased by 3% (95% CI: 2.2–3.1%) as male nest attendance decreased by 10% (Figs 1a and 2a, Table S1^33^). Thus, females ‘partially compensated’ for this decrease (i.e. incubated for 62% of male absence, 95% CI: 56–67%). As female responsibility for the nest increased, their nest attendance increased as well, but less than would be expected under the ‘full compensation hypothesis’ (Figs 1a and 2b, Supplementary Table S2^33^; note that in Fig. 2b hypothetical full compensation is indicated by dashed line and in case of no compensation the points would be parallel to x-axis).

### Daily nest attendance

**Figure 3:**
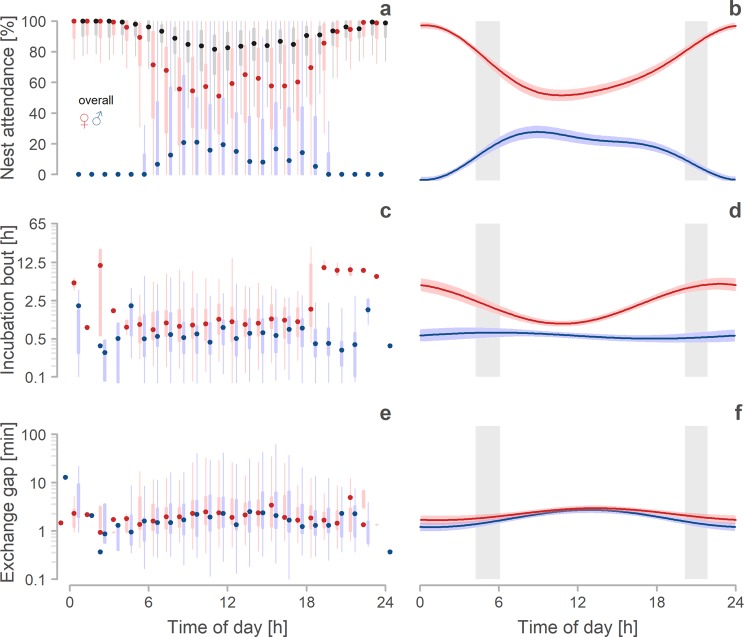
Daily changes in incubation behaviour. (**a**,**c**,**e**) Daily variation in overall (dark grey), female (red) and male (blue) nest attendance (**a**), bout length (**c**) and exchange gap (**e**). Points depict hourly median weighted by sample size for each nest. Thicker lines indicate 25–75th percentiles, thinner lines 25th and 75th percentiles minus or plus 1.5× interquartile range, respectively, or the minimum and maximum value, whichever is smaller. Note that outliers are not depicted. Included are only those hours with complete incubation records (*N* = 7933 hours from 113 nests; median 61 hours per nest, range: 24–482) and complete incubation bouts (*N* = 3184 bouts from 107 biparentally incubated nests; median 20 bouts and exchange gaps per nest, range: 1–297). (**b**,**d**,**f)** Predicted relationships between time of day and nest attendance (**b**), bout length (**d**) and exchange gap length (**f**) according to sex (female in red, male in blue). Lines with shaded areas indicate model prediction with 95% credible intervals based on the joint posterior distribution of 5,000 simulated values from mode outputs (Tables S4, S5 and S8^33^) and generated by the ‘sim’ function in R^90^. Grey bars indicate the period between the earliest and the latest sunrise and sunset during incubation monitoring.

Daily nest attendance mirrored the overall nest attendance (median = 88%, range: 50–98%, N = 191 days from 78 nests) and also decreased by 3.6% (95% CI: 2.1–4.1%) with every 10% decrease in male nest attendance (Supplementary Fig. S1 and Table S3^33^). Daily nest attendance was repeatable in females (0.54, 95%CI: 0.37–0.67), as well as in males (0.7, 95%CI: 0.58–0.8). Daily nest attendance (overall, female or male) was unrelated to the day of the incubation period or day when the nest was started within the breeding season (Table S3^33^). However, overall nest attendance varied strongly within a day, being highest and nearly continuous during the night (median nest attendance between 22:00. and 4:00 was 100%) and lowest during the day (Fig. 3a - in dark grey). Females were almost always the incubating sex at night, and dominated the nest attendance also during the day (Fig. 3a,b; Table S3^33^). Specifically, female nest attendance dropped after sunrise, while male nest attendance peaked after sunrise and also before sunset (Fig. 3a,b; Table S3).

### Incubation bouts and exchange gaps

**Figure 4:**
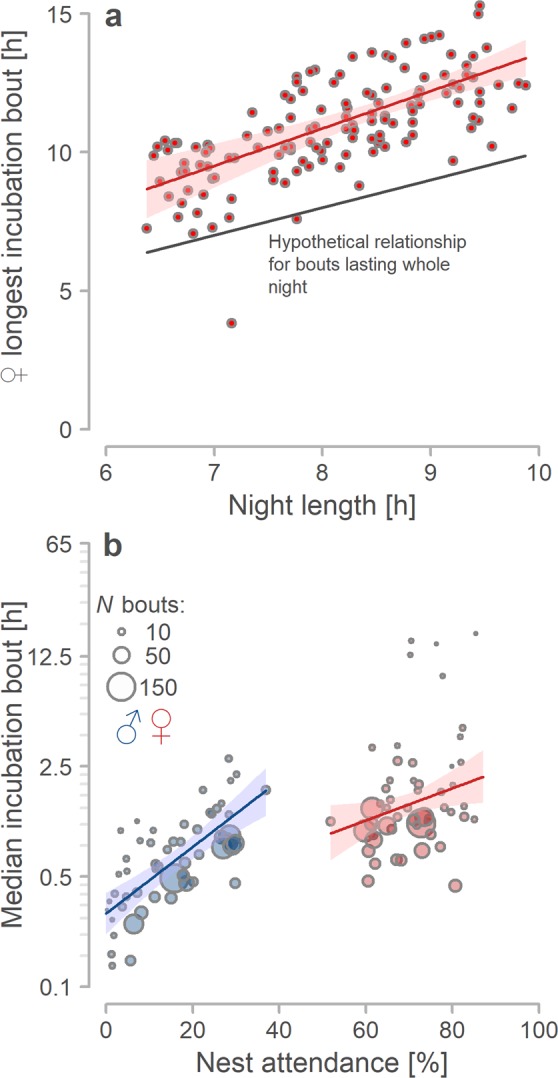
Bout length correlates. (**a**) Longest female incubation bout during a particular night in relation to the night length - defined as time when the Sun is >6° under the horizon. Points indicate bouts (*N* = 133 bouts from 55 nests; median 2 nights per nest, range 1–14 night; included are only bouts with at least 60% of their length in nights). The thick grey line indicates a situation when incubation bouts would last the whole night. (**b**) Median bout length in relation to sex (females in red, males in blue) and nest attendance. Circles represent individual parents, their size the number of days with incubation data. (*N* = 110 parents from 55 biparentally incubated nests with more than two days of continuous recording). (**a**,**b**) Lines with shaded areas indicate model prediction with 95% credible intervals based on the joint posterior distribution of 5,000 simulated based on model outputs (Tables S6 and S7^33^) and values generated by the ‘sim’ function in R^90^.

In biparental nests (i.e. nests where both females and males incubated), incubation bouts lasted 44 minutes (median, range: 1 second –42 hours, *N = *3184 bouts from 107 nests) and varied greatly between and within nests (Fig. 1b, Supplementary Actograms^33^) and especially over the day (Fig. 3c,d); bouts (especially of females) were longer during the night than during the day (Fig. 3c,d). Female incubation bouts lasted 60 minutes (median, range: 1 minute –42 hours; N = 1518) whereas male incubation bouts lasted 32 minutes (median, range: 1 second –7.9 hours; N = 1666). Notably, on average and regardless of time, female bouts were always longer than those of males (Fig. 3c,d; Table S5^33^), although during the daytime females had shorter median incubation bouts than males at 30% of nests (Fig. S3b^33^). Also note that median female and male bout lengths were uncorrelated (Fig. S3b^33^). Overall, incubation bouts were similar across the incubation period and unrelated to the day when the nest started within the breeding season, but note the tendency in males for shorter incubation bouts as the incubation period progressed (Fig. S2, Table S5^33^). Essentially, as the breeding season progressed and nights became shorter, longest night incubation bouts of females shortened too (Fig. 4a, Table S6^33^). Also, median bout length of females and males positively correlated with their nest attendance (Figs 1 and 4b; Table S7^33^).

Exchange gaps lasted 1.9 minutes (median; range 6 seconds –2.5 hours; *N* = 3184 exchange gaps from 107 nests). Length of exchange gaps was unrelated to the day in the incubation period and the day when the nest started within the season. However, exchange gaps fluctuated over the course of the day, being longest in the middle of the day (noon median = 2.49 minutes, range: 0.13–39.5 minutes, interquartile range: 1.32–5.32), and shortest during early mornings (5:00 o’clock median = 1.16 minutes, range: 0.35–19.3 minutes, interquartile range: 0.68–2.05) and evenings (19:00 o’clock median = 1.41 minutes, range: 0.28–87.5, interquartile range: 0.61–2.63; Fig. 3e,f; note that there is only negligible number of exchange gaps during the night). Also, exchange gaps that occurred before female incubation bouts were approximately 24 seconds longer (estimate, 95% CI: 15–35 seconds) than those before male incubation bouts (Fig. 3e,f; Table S8^33^).

## Discussion

Using continuous video monitoring, we quantitatively described incubation rhythms in a central European population of a common Palearctic shorebird, the Northern Lapwing. Our data set allowed us to confirm and reveal three main aspects of Northern Lapwing incubation rhythms. We show that (1) nests varied substantially in overall nest attendance and overall nest attendance strongly correlated with male nest attendance. We further reveal that (2) females partially compensated for the general lack of male nest attendance and that (3) the incubation rhythms varied little over the incubation period and season, but varied strongly over the course of the day. We discuss these three aspects in detail below.

### Overall incubation rhythm

Overall nest attendance was 87% (median; range: 67 to 94%; Fig. 1a), which is in line with findings from other Lapwing populations from the Netherlands and Norway^27–29,31^. Yet, it remains unclear why Northern Lapwings do not incubate more, given that closely related species can achieve higher nest attendance even when incubating uniparentally and in more northerly regions^42,43^. Perhaps breeding in a temperate climate does not require as continuous nest attendance as in other harsher climates (but see temperate species in ref.^34^). Essentially, the between nest variability in nest attendance – which can vary by as much as a 6.5 hours per day – seems huge and is much larger than nest attendance fluctuations known to influence embryo development^32–34^, offspring quality and survival^44–49^ or length of the incubation period^23,32,33,35^.

Moreover, we found that nest attendance positively correlated with the length of incubation bouts (Fig. 4b) and that exchange gaps between incubation bouts were short (median = 1.9 minutes; Fig. 3c). These findings suggest that the departures of a parent from its nest (within its incubation bout) do not trigger their partner to come and incubate^30,50^, although this partner spends most of its time inside the breeding territory and thus usually sees its nest^29,31^. Hence, in Northern Lapwings other (to date unknown) cues drive the decision of parents to return to the nest and incubate. These findings resemble those from other species, albeit in most of those the off-nest partner is far from the nest^7,8,13^.

We also found that male nest attendance varied from 0 to 37% of observed time, with a median of only 13% (mean = 14%; Fig. 1). Such male contributions are lower than in other Northern Lapwing populations (mean: 20% in ref.^28^, 27% in ref.^29^; median: 19% in ref.^31^, 22% in ref.^51^).This difference might be partly driven by the daytime only monitoring of incubation in most previous studies^29,31,51^. If we include only day-light period, male nest attendance in our data rises to 19% (both median and mean). Importantly, the immense variability in male nest attendance is rare among shorebirds^6^. Yet, we know little about the drivers of this between population variability in male contribution to incubation. There is some evidence that the population differences in *Charadrius* plovers correlate with differences in local climate or operational sex ratio of the population^22,23,52^. In non-shorebird species, the population differences in male care (in general) were linked to population differences in the food abundance^53,54^.

The low male nest attendance across Northern Lapwing populations indicates that males either invest into parental care less than females or invest into parental care differently than by incubating, e.g. by guarding the nest (and the female) against predators^29,55^. Importantly, the variation in male nest attendance may reflect the male’s mating status (not recorded in this study), as some Northern Lapwing males tend to have more than one female (reviewed in ref.^56^). Indeed, in other species, the amount of polygyny is linked to the variation in male nest attendance^57,58^. Nevertheless, in Northern Lapwings, the available evidence for this relationship is inconsistent, perhaps because it is based on non-continuous monitoring^27,29,30^.

### Relationship between female and male nest attendance

Notably, we also found that when male nest attendance was low, female nest attendance was higher, but not enough to fully compensate for the male decrease. Hence, with decreasing male attendance, overall and daily nest attendance decreased as well (see Fig. 2 for overall effects, Fig. S1 for daily effects^33^). These findings suggest that, unlike in other species such as geese with uniparental female incubation and close to 100% nest attendance^59^, the Northern Lapwing females have a limited capacity to incubate continuously (i.e. nor our uniparental females, nor Lapwing females in other studies maintained continuous close to 100% nest attendance throughout the incubation period^28,29^). Such limited capacity to incubate continuously is in line with nest attendance of uniparentally incubating shorebirds^24,42^. Although our results are only correlational and thus experiments (e.g. temporal removal of a male^60^) are needed to elucidate these patterns of partial compensation, the findings are in line with previous empirical and theoretical work^61–64^, which suggests that a biparental care will be evolutionary stable only if a parent compensates partially for a reduced parental care of its partner. Such partial compensation is feasible only in environments (like in this temperate Northern Lapwing population) where a decrease in parental care does not necessarily translate into breeding failure, e.g. due to cooling or overheating of eggs^22–24,65^.

### Seasonal and daily variation in incubation rhythms

#### Season

Incubation rhythms were generally stable during the incubation period and season (Tables S3, S5, S8^33^) but varied strongly within a day (Fig. 3). The general lack of variation in Northern Lapwing nest attendance across the incubation period and season contrasts with findings from other species where, for example, incubation bouts lengthen over the incubation period and then shorten just before hatching^13,25,66^. Note that the lack of variation across the incubation period in our study may also reflect a lack of statistical power, that is 5–8 days of incubation data at the start and end of incubation period may still not be enough, in face of within- and between-nest variation. However, we also found that female night bouts shortened as nights shortened with the progressing breeding season. We propose that this seasonal pattern results from daily incubation rhythm of Lapwings where females take nearly sole responsibility for their nest and incubate continuously with one or few long incubation bouts over the whole night. As nights become shorter, the night incubation bouts also shorten – something worth investigating in other species.

#### Daily variation

Overall nest attendance and female nest attendance were highest during the night and lowest during the day; in contrast, males rarely incubated at night and their nest attendance peaked (Fig. 3b) after sunrise and before sunset.

Why do Northern Lapwings incubate so differently during the day and night? Anti-predation strategy does explain variation in incubation rhythms of shorebirds on a comparative scale; species that rely on camouflage when incubating (i.e. are cryptic) have longer incubation bouts than those which do not^6^. Thus, while Northern Lapwings actively attack predators and have short incubation bouts during the day^29,55^, they may minimize number of changeovers on the nest during the night when mammalian predators are more active, leading to long incubation bouts. This may reduce olfactory cues as incubating birds may smell less than unincubated clutch^67^ and will reduce visual cues. Also, poor visibility during the night may prohibit Lapwings from attacking mammalian predators. Perhaps, more importantly, attacking a mammal may not deter it from further searching for the eggs. Indeed, in our population all video-recorded egg predation events occurred during the night and by mammals (mainly by Red fox *Vulpes vulpes* and Stone Marten *Martes foina*; unpublished data).

Apart from anti-predation strategy and predatory risk, the day-night differences in lapwing nest attendance may arise from circadian variation in ambient temperatures and food availability. First, as ambient temperature falls during the night, continuous incubation might be necessary to sustain embryonic development^45,46^. Indeed, also other species (including the uniparental ones) increase their night-time nest attendance^24,68,69^. Second, as food availability might be lower during the night (e.g. due to lower activity of arthropods), Lapwings may prefer daytime foraging and have limited or no need to be off the nest during the night. However, this seems improbable, since Northern Lapwings forage more and with higher food intake during the night^70^. Similarly, other shorebirds have either similar food intake across day and night or higher intake during the night^71^.

The lack of male night nest attendance (Fig. 3a) corresponds with findings from other Lapwing populations^28,30^. We found some night incubation only in 5 out of 55 incubating males (9%) and the five males took care of less than 1% of the nocturnal incubation effort; In Norway males never incubated at night and the Netherlands 10 out 20 males attended the nest at night (50%), but again only with 8% of night attendance. Lack of male night nest attendance (i.e. female-only night incubation) is reported also in related plover species of genus *Pluvialis*^6,34,72^. Still, why do Lapwing males incubate so rarely during the night? One hypothesis suggests that the brighter parent should incubate at night^73^. However, in Northern Lapwings (as well as in other species genus *Vanellus* and *Pluvialis*), sexes are rather similar. If anything, males are the more ornamented sex^74,75^ and hence should incubate during the night, which is not the case. Importantly, the sexual colour dimorphism of Northern Lapwings is similar to sexual colour dimorphism of other *Charadrius* species with predominantly male night incubation^11,76–78^. Alternatively, Northern Lapwing (and *Pluvialis*) males might be less efficient incubators, e.g. warm the eggs to lower temperatures (as is the case in other species^16,18,20,21^), which would favour female to attend the nest during times with lower ambient temperatures, that is during the night^79^. The efficiency of male incubation in Lapwings or other closely related species with female night nest attendance is to date unexplored, but our limited descriptive evidence suggest minimal difference between Lapwing females and males in incubation temperatures (Supplementary Actograms^33^).

Notably, we depicted the distribution of male nest attendance across the day with peaks after sunrise and before sunset (Fig. 3a). A similar (but role-reversed) situation seems to be present in some Kentish plover (*Charadrius alexandrinus*) populations^22^. We speculate that by incubating after sunrise and before sunset males may allow females to replenish their energy stores after and before long night incubation bouts. We thus propose testing whether females lacking male contribution to care will weigh less and incubate less in the morning, at the end of the day or at the end of the incubation period than females with male contributions to incubation^80^.

## Conclusion

To conclude, with continuous monitoring of 113 Northern Lapwing nests we demonstrate (a) how male contribution to incubation links to the substantial within population variability in incubation rhythms, and that (b) the incubation rhythms were generally stable over the days, but strongly fluctuated across 24 h-day, being modulated not only by day and night, but also by sunrise and sunset. The next step is to experimentally investigate what drives the variation in male incubation and whether the sunrise and sunset driven modulations (or even other modulations) of the circadian incubation rhythms are common also in other populations, species and environments.

## Methods

### Data collection

In April-May of 2015 and 2016 we monitored incubation of Northern Lapwings in České Budějovice basin, Doudlebia, Czech Republic (49.25°N, 14.08°E), on approximately 40 square kilometres of agricultural land. We searched for nests by systematically scanning fields and meadows with telescopes, or by walking through areas with high nest densities. If a nest was found during laying (i.e. with a lower clutch size then during later nest visits), we estimated its start of incubation by assuming that females laid one egg per day and started incubation when the clutch was complete (usually four, rarely three eggs). If a nest was found with a full clutch, we estimated its start of incubation based on the median height and angle at which the eggs floated in water^81^ and assuming an incubation period of 27 days (unpublished data).

We monitored incubation with a custom designed video recording system (Jan Petrů, Czech Republic), consisting of an external lens (Ø 2 cm, length 4 cm) mounted on a ~30 cm long twig and placed 1.5 meters from the nest in a southward direction to minimize the time the lens faced the sun, which would have overexposed the videos and made individuals hard to recognize. Infra-red light (within the lens) of 10 out of 15 systems was used to record the night time nest attendance. The digital recorder stored videos in 10–15 frames per second in 640 × 480 pixels resolution for about four days. The system was powered by a 12-V, 44-Ah battery buried together with the recorder (in a waterproof case) under the ground (Supplementary Picture S1^33^). Two to three people installed the equipment, which took about 10 minutes per nest. To minimize the number of visits to a particular site, we often equipped several nests at a time. Note that parents (regardless of the sex) returned to the nest within 46 minutes after installation (median; for females: median = 45 minutes, 2.5th quantile = 9 minutes, 97.5th quantile = 3.7 hours; for males: median = 56 minutes, 2.5th quantile = 22 minutes, 97.5th quantile = 4.3 hours). Thus, the camera could have influenced behaviour of some sensitive individuals.

In addition, at six of the video-recorded nests, we also recorded nest temperature and surface temperature next to the nest using MSR 145B5 dataloggers (0.1 °C accuracy) and small external probes placed among the eggs^6,13^.

All field procedures were performed in accordance with the relevant guidelines and regulations, and approved by the institutional committee, based on the institutional accreditation No. 63479/2015-MZE-17214 of Ministry of Agriculture of the Czech Republic.

### Extraction of incubation behaviour

We extracted incubation behaviour from video recordings in AVS Media Player (http://www.avs4you.com/AVS-Media-Player.aspx) by noting the date and time (to the nearest second) when a bird came to the nest (both legs in the nest) or left the nest. We thus define incubation as both sitting on the eggs (warming) or standing above them (turning them or shading them from direct sunlight). We distinguished females and males via individual and sex-specific plumage traits such as crest length, or the extent of melanin ornaments on the face and breast^74^ – a technique widely used to distinguish female and male of this species^28,56,82–85^ because the overlap of crest length or breast colour between the sexes is minimal^74^.

We further noted any disturbance caused by the field team, agricultural work, general public or interaction with other animals (note that only bouts with disturbance from the field team were excluded from the analyses). Bouts with technical difficulties and with low visibility, when parents were hard to recognize (e.g. during direct sunlight or heavy rains), were classified as uncertain and excluded from the analyses (<1% of recorded time; see Supplementary Actograms for details, raw incubation data and extracted incubation bouts^33^).

### Definition of incubation variables

We defined nest attendance as the proportion of time a nest was actually incubated by one of the parents, i.e. a parent being on the nest (including shading of eggs), which excludes incubation recesses. Specifically, ‘overall nest attendance’ indicates attendance for the whole time a nest was monitored; ‘daily nest attendance’ indicates attendance for a particular day and nest; ‘hourly nest attendance’ indicates attendance for a particular hour in a particular day and nest. Female or male nest attendance denotes proportion of incubation by a particular sex during a respective time interval (e.g. overall, day, hour or incubation bout).

Furthermore, we define incubation bouts as the total time allocated to a single parent (i.e. the time between the arrival of a parent at the nest and its departure, followed by the incubation of its partner) and exchange gaps as the time between the departure of one parent from the nest and the return of its partner. Note that incubation bouts include also incubation recesses and that an incubation bout of one parent is an off-nest bout of the off-duty parent.

Last, responsibility at each nest indicates a proportion of monitored time taken by all incubation bouts of a given parent (i.e. the sum of all incubation bouts of a given parent divided by the total observation time).

### Sample sizes

We monitored 107 nests (46 in 2015 and 61 in 2016) for a median 3 days (range: 1–7 days). Because we caught and individually marked only 5% of the monitored parents, we cannot rule out the possibility that some parents were monitored during multiple breeding attempts. However, we believe this was rare because (a) in both years we observed only up to 25% of our breeding population (~200 nests) and because (b) out of 73 individuals caught during 2014–2017, we observed only 10% during subsequent years. Thus, the repeated sampling, if any, is rare and hence its consequence on our analysis minimal.

To increase the number of nests monitored for nearly the whole incubation period, we included another 6 nests from a different study (also from the Czech Republic, 49.90°N, 15.98°E) monitored for 14 days (median; range: 8–22 days^6,34^; the Lapwing part of the study collected the incubation data between 2009 and 2011 using the same method as we do here^6,34^).

However, not all incubation data and nests were suitable for all analyses. For the analyses of overall nest attendance, we used only nests with at least two complete days of recorded incubation (*N* = 60 nests with median of 3 days per nest; range: 2–20 days). For the analyses of daily nest attendance, we used only nests with at least one day of recorded incubation (*N* = 191 days from 78 nests with median of 2 days per nest; range: 1–20 days). For both, nest level and daily nest attendance data, we used only days monitored for more than 90% of the day. For the analyses of hourly nest attendance, we used only nests with at least 24 hours of recording and only hours with continuous incubation recording (*N = *113 nests with a median of 61 hours, range: 24–482 hours). We used the same nests (but excluding uniparental ones) for the analyses of incubation bouts and exchange gaps (*N* = 107 nests with median of 20 incubation bouts and exchange gaps per nest, range: 1–297 bouts and exchange gaps).

### Statistical analysis

All procedures were performed in R version 3.3.0^86^. General linear models were fitted using the ‘lm’ function^86^ and mixed-effect models using the ‘lmer’ function from the ‘lme4’ R package^87^. For each model parameter we report effect size and model predictions as medians and the Bayesian 95% credible intervals (95%CI) represented by 2.5 and 97.5 percentiles from the posterior distribution of 5 000 simulated or predicted values obtained by the ‘sim’ function from the ‘arm’ R package. We estimated the repeatability of female and male daily nest attendance (i.e. between-days stability in division of incubation) using the’rpt’ function from the ‘rptR’ R package, restricted maximum likelihood method (REML), gaussian model, and 5 000 bootstrapped runs^88^.

All continuous predictors (except for time) were z-transformed (mean centered and divided by standard deviation)^89^. Whenever we tested for the rhythmicity in a response (period of 12 h or 24 h), we transformed time to radians (2*time *π*/*period of interest) and then fitted the sinus and cosinus of radians^6^. Where appropriate, models were weighted by the square root of monitored time (e.g. number of days or proportion of monitored time within the day; see Supplementary Tables for details).

#### Overall nest attendance

To explain between-nest variation in overall nest attendance we fitted overall nest attendance (%) as a response and male nest attendance as a predictor (Table S1). To explore the relationship between nest attendance and responsibility of each parent, we fitted nest attendance as a response, and sex of the parent in interaction with its responsibility for the nest (%) as predictors (Table S2). As female and male nest attendance and responsibility at a given nest may not be independent of each other, we further fitted nest identity as a random intercept.

#### Daily nest attendance

We investigated the correlates of variation in between- and within- day nest attendance with two mixed-effect models with gaussian response variable. In the first (Table S3), we specified daily nest attendance as a response and three predictors: the number of days from the beginning of the incubation, i.e. day in incubation period (‘Day of incubation‘), day when incubation started (‘Start of incubation’) and male daily nest attendance (‘Male attendance’). Furthermore, we specified nest identity as a random intercept and male daily nest attendance as a random slope. In the second (Table S4), we specified hourly nest attendance as a response. As hourly nest attendance of female and male were inversely correlated (r_s_ = −0.72), to ensure independence of data points we randomly sampled for each hour one sex so that each hour had only one sex associated with it. We then fitted sex in interaction with time transformed (in the above described manner) with 12 hour periodicity (’12 time’), as well as in interaction with time transformed with 24 hour periodicity (’24 time’). ‘Day in season’ and parent identity nested in nest identity were fitted as random intercepts and time predictors were fitted as random slopes.

#### Incubation bouts and exchange gaps

To investigate variation in incubation bouts and exchange gaps we fitted four models. In the first model (Table S5), we fitted incubation bout (ln-transformed) as a response and sex, ‘Day of incubation’ and ‘Start of incubation’, all in interaction with time (with 24 hour rhytmicity) as predictors. To eliminate temporal autocorrelation, we included also the length of the previous incubation bout (ln-transformed). Nest identity and day in season were fitted as random intercepts. As the influence of time may differ between the nests or over the season, we further fitted time and day in incubation period as random slopes.

In the second model (Table S6), we fitted the longest female night bout (ln-transformed) as a response. Such bout had at least 60% of its length in the night (i.e. when sun was >6° below the horizon). We specified the length of the night as a predictor, the nest identity as a random intercept and the length of the night as a random slope.

In the third model (Table S7), median bout per parent and nest (ln-transformed) was fitted as a response variable and overall nest attendance per parent in interaction with sex as predictors. Nest identity was used as a random intercept. In the fourth model (Table S8), we fitted the length of exchange gap (ln-transformed) as a response and sex,’Day of incubation’ and ‘Start of incubation’, all three also in interaction with time (24 hour rhytmicity) as predictors. Nest identity was specified as a random intercept and ’24 time’ and ‘Day of incubation’ as random slopes.

Note that in some models we attempted to use more complicated random structures, but the models never converged (for further details see legends in Supplementary Tables^33^).

## Supplementary information

Supplementary information
