# Supplementary material for: Diversity of incubation rhythms in a facultatively uniparental shorebird – the Northern Lapwing
Source: Sci Rep. 2019 Mar 18;9:4706. doi: 10.1038/s41598-019-41223-z (PMC6423287; doi:10.1038/s41598-019-41223-z)
Supplement: Supplementary file 1 — Supplementary information [file 41598_2019_41223_MOESM1_ESM.pdf]

SUPPLEMENTARY INFORMATION for

Diverse incubation rhythms in a facultatively uniparental shorebird – the northern lapwing

Martin Sládeček sladecek@fzp.czu.cz, Eva Vozabulová, Miroslav E. Šálek and Martin Bulla

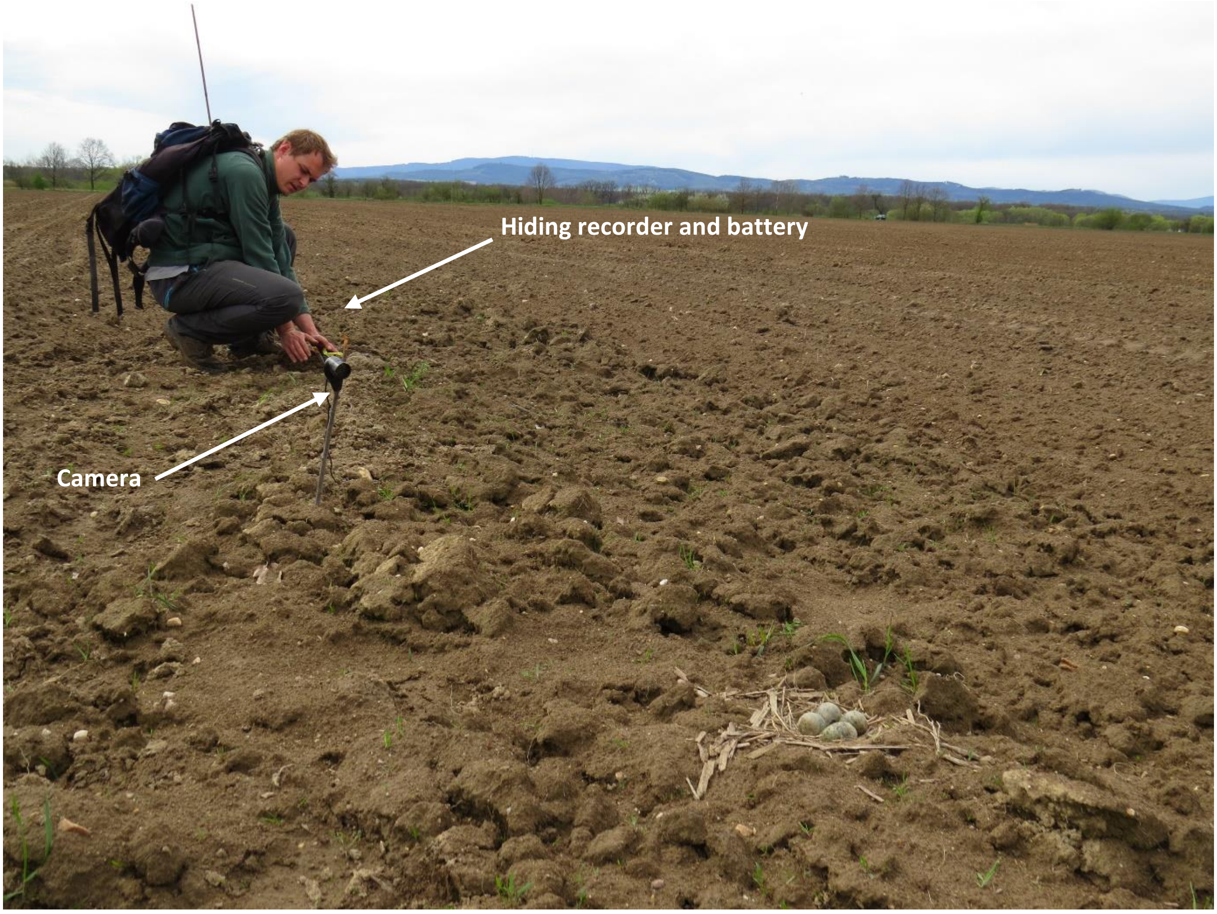

Picture S1. Example of video-recording equipment at the nest. The camera lens monitors the nest, its cable is hidden under the ground and so is the recording equipment and the battery.

Table S1 | Overall nest attendance in relation to male nest attendance

Response	Effect	Estimate	95% CI Lower	Upper
Overall nest attendance	Intercept	0.817	0.799	0.835
(proportion of monitored time)	Proportion of male incubation	0.318	0.224	0.412

The posterior estimates (medians) of the effect sizes with the 95% credible intervals (CI) from a posterior distribution of 5,000 simulated values generated by the 'sim' function in R[1]. Included are only nests with at least two days of incubation data and days with at least 90% of recording (*N* = 60 nests). Results of this Gaussian model were weighted by square root number of monitoring days.

Table S2 | Nest attendance in relation to responsibility and sex of the parent

Response	Effect type	Effect	Estimate	95% CI Lower	Upper
Nest attendance	Fixed	Intercept	0.212	0.168	0.257
(proportion per parent)		Sex	-0.213	-0.259	-0.166
		Incubation responsibility	0.618	0.564	0.672
		Sex x Incubation responsibility	0.302	0.218	0.385
	Random	Nest (Intercept)	4%		
	(variance)	Residual	96%		

The posterior estimates (medians) of the effect sizes with the 95% credible intervals (CI) from a posterior distribution of 5,000 simulated values generated by the 'sim' function in R[1]. Variance components were estimated by the 'lmer' function in R[2]. Included are only nests with at least two days of incubation data and days only days monitored for more than 90% of day (*N* = 120 parents from 60 nests). Results of this Gaussian model were weighted by square root number of monitoring days.

Table S3 | Daily nest attendance in relation to male nest attendance, incubation period and season.

Response	Effect type	Effect	Estimate	95% CI Lower	Upper
Nest attendance	Fixed	Intercept	0.867	0.857	0.876
(proportion per day)		Day of incubation	0	-0.009	0.009
		Start of incubation	0.002	-0.008	0.011
		Male attendance	0.043	0.033	0.053
		Day of incubation x Start of incubation	0.003	-0.006	0.012
	Random	Nest (Intercept)	12%		
	(variance)	Male attendance	19%		
		Residual	69%		

The posterior estimates (medians) of the effect sizes with the 95% credible intervals (CI) from a posterior distribution of 5,000 simulated values generated by the 'sim' function in R[1]. Variance components were estimated by the 'lmer' function in R[2]. Included are only days monitored for more than 90% of day (*N* = 191 days from 78 nests). Continuous predictors and the random slope were z-transformed (mean-centred and divided by SD). The Gaussian model was weighted by square root of monitored time within day (proportion). Note that a model containing also random slope of 'Day of incubation' period did not converge. However, including incubation period as random slope instead of male attendance yielded similar estimates (that is the lack of 'Incubation period' random slope does not seem to overestimate current results).

Table S4 | Hourly nest attendance in relation to time of day.

Response	Effect type	Effect	Estimate	95% CI	
				Lower	Upper
Nest attendance (proportion per hour)	Fixed	Intercept	0.718	0.7	0.736
		Sex (M)	-0.571	-0.596	-0.545
		Sin (24 time)	-0.012	-0.027	0.003
		Cos (24 time)	0.218	0.196	0.242
		Sin (12 time)	0.021	0.008	0.035
		Cos (12 time)	0.027	0.01	0.044
		Sin (24 time) x Sex (M)	0.029	0.008	0.05
		Cos (24 time) x Sex (M)	-0.357	-0.39	-0.325
		Sin (12 time) x Sex (M)	-0.039	-0.058	-0.019
		Cos (12 time) x Sex (M)	-0.071	-0.095	-0.047
	Random (variance)	Nest (Intercept)	3%		
		Sin (24 time)	3%		
		Cos (24 time)	6%		
		Sin (12 time)	4%		
		Cos (12 time)	6%		
		Sex within nest (Intercept)	7%		
		Sin (24 time)	2%		
		Cos (24 time)	10%		
		Sin (12 time)	1%		
		Cos (12 time)	4%		
		Day in season (Intercept)	0%		
		Sin (24 time)	0%		
		Cos (24 time)	0%		
		Sin (12 time)	0%		
		Cos (12 time)	0%		
		Residual	53%		

The posterior estimates (medians) of the effect sizes with the 95% credible intervals (CI) from a posterior distribution of 5,000 simulated values generated by the 'sim' function in R[1]. Variance components were estimated by the 'lmer' function in R[2]. As female and male attendance within given hour are not independent, for each hour we randomly sampled the sex, whose nest attendance we then used as a Gaussian response (*N* = 7933 fully monitored hours from 113 nests; 3902 hours of female nest attendance and 4031 hours of male attendance). This procedure also dramatically reduced temporal autocorrelation of residuals. To further account for non-independence of data points, 'Sex' nested in 'Nest' (i.e. specifying bird ID) and day in season where fitted as random intercepts and 'time' as a random slope. 'Time' was transformed to radians (2*time * π/period of interest – 12 or 24h) and fitted as sine and cosine of radians. Note that we tested not only for circadian 24h-rhythmicity, but also for 12-hour rhythmicity in nest attendance, as based on our observations sex-specific nest attendance changes after sunrise and before sunset. Also, simplifying the random structure of the model by omitting random slopes yielded similar estimates.

Table S5 | Length of incubation bouts in relation to male nest attendance, incubation period and season.

Response	Effect type	Effect	Estimate	95% CI Lower	Upper
Bout length	Fixed	Intercept	4.7	4.56	4.84
(hour)		ln (Length of previous bout)	0.062	0.018	0.108
		Start of incubation	-0.137	-0.271	0.002
		Day of incubation	-0.065	-0.161	0.036
		Sex (M)	-1.346	-1.46	-1.228
		Sin (time)	-0.261	-0.369	-0.15
		Cos (time)	0.8	0.651	0.953
		Cos (time) × Start of incubation	-0.104	-0.228	0.019
		Sin (time) × Start of incubation	0.018	-0.083	0.112
		Sin (time) × Day of incubation	-0.047	-0.122	0.028
		Cos (time) × Day of incubation	-0.06	-0.165	0.041
		Sin (time) × Sex (M)	0.391	0.282	0.493
		Cos (time) × Sex (M)	-0.791	-0.966	-0.611
		Start of incubation × Sex (M)	0.023	-0.056	0.103
		Day of incubation × Sex (M)	-0.14	-0.216	-0.061
	Random	Day in season (intercept)	2%		
	(variance)	Sin (time)	1%		
		Cos (time)	3%		
		Nest (intercept)	10%		
		Sin (time)	5%		
		Cos (time)	3%		
		Day of incubation	0%		
		Residual	76%		

The posterior estimates (medians) of the effect sizes with the 95% credible intervals (CI) from a posterior distribution of 5,000 simulated values generated by the 'sim' function in R[1]. Variance components were estimated by the 'lmer' function in R[2]. Only complete incubation bouts were used (*N* = 3184 bouts from 107 biparentally incubated nests). To approach normality of residuals, 'Bout length' was ln-transformed and fitted as Gaussian response. Continuous predictors were z-transformed (mean-centred and divided by SD). 'Time' was transformed to radians (2*time *π / 24h) and fitted as sine and cosine of radians. To eliminate temporal autocorrelation in residuals, we also fitted 'Length of previous bout'. To further account for non-independence of data points, 'Nest' and 'Day in season' where fitted as random intercepts and 'time' and 'Day of incubation' (within 'Nest') as random slopes. Note that models containing also random intercept for 'Sex' nested in 'Nest' (i.e. specifying bird ID) did not converge.

Table S6 | Length of female night bout in relation to length of the night.

Response		Effect	Estimate	95% CI Lower	Upper
Bout length	Fixed	Intercept	0.026	-3.919	3.946
(hour)		Night length	1.351	0.899	1.815
	Random	Nest (Intercept)	97%		
	(variance)	Night length	1%		
		Residual	1%		

The posterior estimates (medians) of the effect sizes with the 95% credible intervals (CI) from a posterior distribution of 5,000 simulated values generated by the 'sim' function in R[1]. Variance components were estimated by the 'lmer' function in R[2]. For each female, we included her longest incubation bout during given night, and only bouts with at least 60% of their length in nigh; night defined as sun being > 6° under the horizon (*N* = 133 bouts from 55 nests). Bout length was fitted as Gaussian response. To account for non-independence of data points, 'Nest' was fitted as random intercept and 'Night length' as random slope

Table S7 | Median bout length of a parent in relation to sex and nest attendance of a particular parent.

Response		Effect	Estimate	95% CI Lower	Upper
Median incubation bout of a parent [h]	Fixed	Intercept	2.81	1.329	4.265
		Sex (M)	0.032	-1.534	1.651
		Nest attendance	2.34	0.328	4.461
		Sex (M) x Nest attendance	2.488	-0.629	5.507
	Random	Nest (Intercept)	18%		
	(variance)	Residual	82%		

The posterior estimates (medians) of the effect sizes with the 95% credible intervals (CI) from a posterior distribution of 5,000 simulated values generated by the 'sim' function in R[1]. Variance components were estimated by the 'lmer' function in R[2]. Included are only nests monitored during both day and night time (*N* = 71 nests). 'Median incubation bout of a parent' was ln-transformed and fitted as Gaussian response. As female and male bouts from the same nest are unlikely independent, we fitted 'Nest' as random intercept. Note, the results of this Gaussian model are weighted by square root number of monitoring days.

Table S8 | Exchange gap length in relation to sex, time of day, incubation period, and season.

Response	Effect type	Effect	Estimate	95% CI Lower	Upper
Exchange gap	Fixed	Intercept	0.802	0.7	0.908
(min)		Sex (M)	-0.213	-0.308	-0.118
		Start of incubation	-0.064	-0.165	0.034
		Day of incubation	-0.04	-0.145	0.068
		Sin (time)	-0.1	-0.175	-0.028
		Cos (time)	-0.257	-0.391	-0.121
		Cos (time)x Sex (M)	-0.139	-0.283	0.002
		Sin (time) x Sex (M)	-0.005	-0.094	0.083
		Day of incubation x Sex (M)	-0.026	-0.089	0.04
		Start of incubation x Sex (M)	0.015	-0.051	0.08
	Random	Nest (Intercept)	6%		
	(variance)	sin(rad)	1%		
		cos(rad)	10%		
		Day of incubation	8%		
		Residual	75%		

The posterior estimates (medians) of the effect sizes with the 95% credible intervals (CI) from a posterior distribution of 5,000 simulated values generated by the 'sim' function in R[1]. Variance components were estimated by the 'lmer' function in R[2]. Same dataset as for analysis of incubation bout length (Table S5) was used (*N* = 3184 exchange gaps from 107 biparentally incubated nests). Response variable 'Exchange gap' was ln-transformed and fitted as Gaussian response. Continuous predictors (except for 'time') were z-transformed (mean-centred and divided by SD). 'Time' was transformed to radians (2*time * π/ 24h) and fitted as sine and cosine of radians. To further account for non-independence of data points, we fitted 'Nest' as a random intercept and 'time' as a random slope. Note that models containing also random intercept of 'Sex' nested in 'Nest' (i.e. specifying bird ID) did not converge.

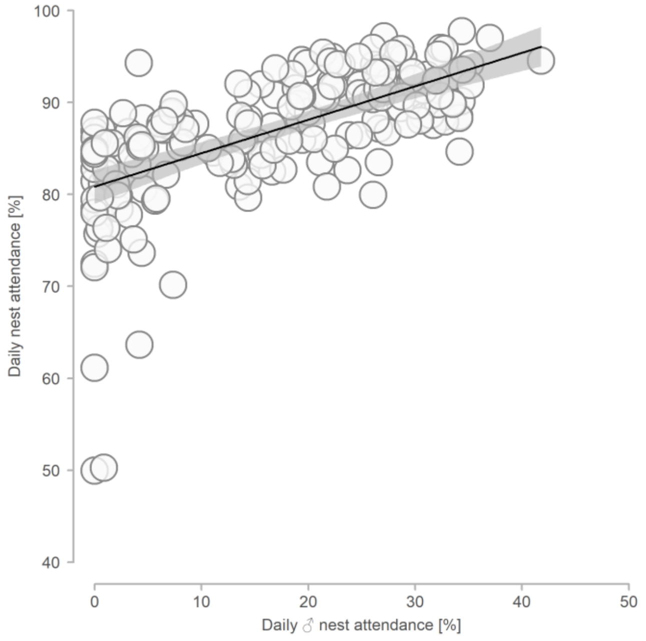

Figure S1. Overall daily nest attendance in relation to male daily nest attendance. Circles indicate individual days (*N* = 191 days from 78 nests; included are only days monitored for more than 90% of day. Line with shaded area indicates model prediction with 95% credible intervals based on the joint posterior distribution of 5,000 simulated values based on the model output (Table S3) and generated by the 'sim' function in R[1].

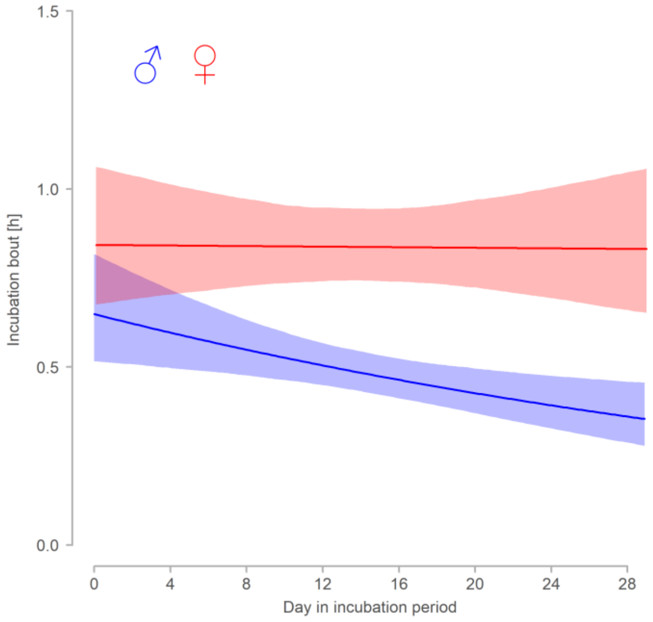

Figure S2. Mean incubation bout length in relation to incubation period and sex. Colour indicates sex (females in red, males in blue), lines with shaded areas indicate model prediction with 95% credible intervals based on the joint posterior distribution of 5,000 simulated values based on the model output (Table S5) and generated by the 'sim' function in R[1] (*N* = 3184 bouts from 107 biparentally incubated nests; only complete bouts were used).

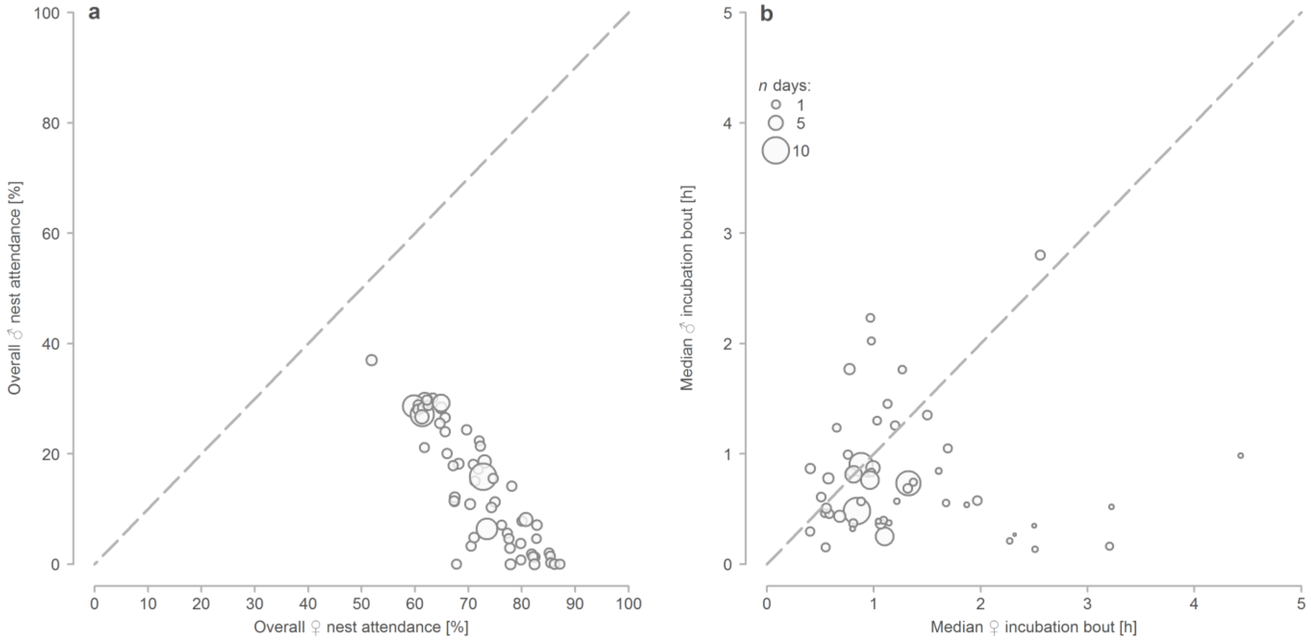

Figure S3. Relationship between female and male incubation. a, Female nest attendance in relation to male nest attendance. **b**, Median length of female incubation bout in relation to median length of male incubation bout (note that only daylight incubation data are included). **a, b**, Circles represent individual nests and their size number of days with incubation data; included are only nests with at least two days of incubation data and days with at least 90% of recording (*N* = 60 nests). Dashed line indicates perfect positive correlation, points above the line nests with male bouts longer than those of females and points below the line nests with female bouts longer than those of males.

REFERENCES

1 Gelman, A. & Su, Y.-S. arm: Data Analysis Using Regression and Multilevel/Hierarchical Models. R package version 1.8-6., http://CRAN.R-project.org/package=arm (2015).

2 Bates, D., Maechler, M., Bolker, B. & Walker, S. Fitting Linear Mixed-Effects Models Using lme4. *J Stat Softw* **67**, 1-48 (2015).
